# Evaluation of the Impact and Satisfaction of Creating a Mother–Child Educational Podcast by University Nursing Students

**DOI:** 10.3390/nursrep16090306

**Published:** 2026-08-28

**Authors:** Sergio Martínez-Vázquez, Rocío Adriana Peinado-Molina, María Antonia Díaz-Ogállar, Antonio Hernández-Martínez, Juan Miguel Martínez-Galiano

**Affiliations:** 1 Department of Nursing, University of Jaén, 23071 Jaen, Spain; svazquez@ujaen.es (S.M.-V.); maogalla@ujaen.es (M.A.D.-O.); jgaliano@ujaen.es (J.M.M.-G.); 2 Consortium for Biomedical Research in Epidemiology and Public Health (CIBERESP), 28029 Madrid, Spain; 3 University Hospital of San Agustin, 23700 Linares, Spain; 4 Department of Nursing, Physiotherapy and Occupational Therapy, University of Castilla-La Mancha, 13071 Ciudad Real, Spain; antonio.hmartinez@uclm.es; 5 Research Group on Care and Evidence-Based Practice, Castilla-La Mancha Institute for Health Research (IDISCAM), Castilla-la Mancha, 45071 Madrid, Spain

**Keywords:** podcast, nursing education, students, nursing, radio

## Abstract

**Aim:** To evaluate the effect of a student-generated podcast intervention focused on maternal–child health topics on nursing students’ self-perceived knowledge, educational satisfaction, and gameful engagement. **Introduction:** Health education is essential for promoting healthy lifestyles and preventing disease, making the acquisition of effective communication skills vital for nursing undergraduates. While digital audio media and podcasts are increasingly popular pedagogical tools, traditional applications rely on passive listening. Student-generated podcast production represents an active learning approach that may enhance engagement, communication skills, and content synthesis. **Design:** Quasi-experimental study without randomization. **Methods:** The intervention was conducted across a 4-week framework comprising 8 contact hours of faculty guidance alongside independent teamwork. First-year nursing students organized into small collaborative groups (3–4 peers) received initial training on digital audio pedagogy, metadata, and production. Teams developed 5-to-7-min podcast capsules addressing eight evidence-based maternal–child health themes. Faculty provided structured formative checkpoints for script clinical validation and technical audio editing guidance. All completed podcasts were evaluated using a 100-point rubric assessing scientific accuracy (35%), communication clarity (25%), narrative engagement (20%), and technical quality (20%); thirty top-scoring episodes were selected for public radio broadcasting. Pre-and post-intervention self-administered questionnaires measured self-perceived knowledge across all eight topics, educational satisfaction, and gameful experience via the validated 27-item Gameful Experience Scale (GAMEX). **Results:** A total of 130 valid questionnaires were analysed at each assessment point. Self-perceived knowledge was higher at the post-intervention assessment across all eight topics (all Holm-adjusted *p* < 0.001). After Holm correction, general podcast satisfaction (adjusted *p* = 0.036), fulfilment of expectations (adjusted *p* = 0.036), recommendation to friends or family (adjusted *p* < 0.001), perceived importance of podcasting for maternal health (adjusted *p* = 0.036), and perceived ease of setting up a podcast (adjusted *p* = 0.005) remained statistically significant. Creative Thinking, Mastery, and the overall GAMEX score were nominally higher before multiplicity adjustment, but none remained statistically significant after Holm correction (adjusted *p* = 0.126, 0.234, and 0.158, respectively). **Conclusions:** Student-generated podcasting was associated with higher self-perceived knowledge and favourable appraisal on several educational outcomes. GAMEX differences were exploratory after Holm correction.

## 1. Introduction

Health education (HE) is essential to promote healthy lifestyles and prevent diseases [1]. Therefore, nursing students must acquire health education skills during their training [2].

Health education in the perinatal stage has been shown to have a positive impact on preparing women for childbirth, as well as improving obstetric and neonatal outcomes, such as fewer cesarean sections or higher breastfeeding rates, among others [3,4,5]. It helps women learn about pregnancy, prenatal care, childbirth, newborn care, and the postpartum period, promoting a healthy and safe motherhood [6,7]. Radio is a universally accessible health communication tool. In developing nations, 75% of households have radio access, and traditional AM/FM stations historically accounted for 86% of listening time among adults aged 25–54 [8]. Delivering health promotion and therapeutic advice via broadcast audio has consistently demonstrated strong cost-effectiveness and audience reach [9,10,11].

More recently, the rise in podcasting has transformed audio-based health education. Driven further by post-pandemic media shifts, global platforms such as Spotify now reach over 761 million monthly active users, including 293 million premium subscribers and a library of 100 million tracks, with worldwide podcast listenership expected to top 584 million [12]. In Spain, monthly podcast consumption has reached approximately 60% of the general population [13]. Through role-play, creative storytelling, and peer feedback, student-generated podcasting transforms passive content delivery into an immersive, active learning task. By offering student agency and structured creative goals, this design process induces a gameful state (GAMEX) despite the absence of explicit gamification mechanics like points or leaderboards. Gamification and advances in educational technologies are having a transformative impact on nursing training [14,15,16]. The European Higher Education Area (EHEA) recommends the use of new educational methodologies, in addition to the evaluation of student satisfaction [17,18], highlighting the podcast format among these methodologies [19]. As a result, radio, transmitted through podcasts, has proven to be effective as a learning method for nursing students [20,21,22,23,24,25].

In recent years, digital audio media and podcasting have gained momentum in health professions education as flexible, technology-enhanced pedagogical tools. While traditional applications in undergraduate nursing education have relied on passive, teacher-led audio resources to reinforce lecture material, contemporary paradigms advocate for engaging students directly as active content creators. Student-generated podcast production encourages clinical reasoning, collaborative communication, and deeper content synthesis. However, empirical research evaluating student-created podcasts specifically within foundational maternal–child nursing modules remains sparse. To address this gap, this study compared self-perceived knowledge, educational satisfaction, and gameful engagement before and after a student-generated podcast intervention focused on maternal–child health topics.

Participation in podcast creation would lead to positive baseline-to-post-intervention shifts in students’ self-perceived topical knowledge (H1), result in high levels of educational satisfaction and positive appraisal of podcasting as a health communication modality (H2), and induce a gamied experience as evidenced by elevated GAMEX scores across its six core dimensions (H3).

## 2. Materials & Methods

### 2.1. Design and Subject Selection

This was an uncontrolled, non-randomized, quasi-experimental pre–post educational study. The intervention was delivered to a single cohort of first-year nursing students. Anonymous self-administered questionnaires were completed before and after the intervention. No individual identifier or linking code was collected; therefore, baseline and post-intervention responses could not be matched at the individual level. Consequently, the two assessment points were analysed as anonymous, unlinked samples drawn from the same eligible cohort. This was an educational intervention outside the scope of the training program for the intervention under study. It was conducted at the University of Jaén (Spain) during the 2024–2025 academic year with a sample of students enrolled in the first year of the Bachelor’s Degree in Nursing. First-year nursing students were selected because maternal–child health is a foundational component of their early curriculum. Implementing an active digital media task during their first year establishes core health communication skills, peer collaboration habits, and digital literacy early in their professional education. Inclusion criteria included all students over 18 years of age who wished to participate. Students who voluntarily declined to participate or did not fully complete the questionnaire were excluded. The sample size was estimated a priori using OpenEpi, version 3.01, through the module for comparing two independent proportions and applying the Fleiss method with continuity correction. The reference outcome was the proportion of students expected to report high or very high satisfaction with the activity. Proportions of 50% at the pre-intervention assessment and 70% at the post-intervention assessment were assumed, corresponding to an absolute difference of 20 percentage points. With a two-sided 95% confidence level, 80% statistical power, and a 1:1 ratio between assessment samples, at least 104 valid questionnaires were required at each assessment point. A total of 130 pre-intervention questionnaires and 130 post-intervention questionnaires were analysed; therefore, the planned sample-size requirement was achieved and exceeded.

### 2.2. Educational Intervention: Podcast Creation Development

An educational intervention was carried out in two phases across a 4-week period involving 8 total contact hours of faculty guidance. The first phase consisted of recruiting nursing undergraduate students from the University of Jaén who were interested in participating. They received information about the project and its phases. After their acceptance, students were organized into collaborative groups of 3 to 4 peers and received training on how to prepare a podcast, covering aspects such as structure (episode, title, description, audio, intro and outro, metadata, image or cover, distribution platform, social media feed, transcripts) and presentation. This allowed them to facilitate distribution, identification, and consumption by the audience. Subsequently, they addressed the topic by presenting relevant examples in this field. After this activity, they were asked to complete an initial self-administered questionnaire. In a second phase, and with the help of university nursing faculty through two structured formative checkpoints (script review for clinical accuracy and technical audio editing guidance), the content was worked on, reviewing the literature and clinical practice guidelines, and developing the podcast capsules (Podcast capsules were defined as short-form, structured digital audio recordings (target duration 5–7 min) focusing on a single clinical topic to deliver concise, evidence-based health guidance), establishing eight well-defined themes:

1. Healthy Lifestyle Habits and Unhealthy Habits During Pregnancy.
2. Proper and Balanced Nutrition. Physical Activity and Exercise During Pregnancy.
3. Expectations and Birth Plan.
4. Relaxation During Labor.
5. Expectations and Feeding of the Baby.
6. Sleep Hygiene During the Postpartum Period.
7. Support Network: Family and Partner During Parenting.
8. Self-Esteem and Personal Relaxation During Parenting.

**Figure 1:**
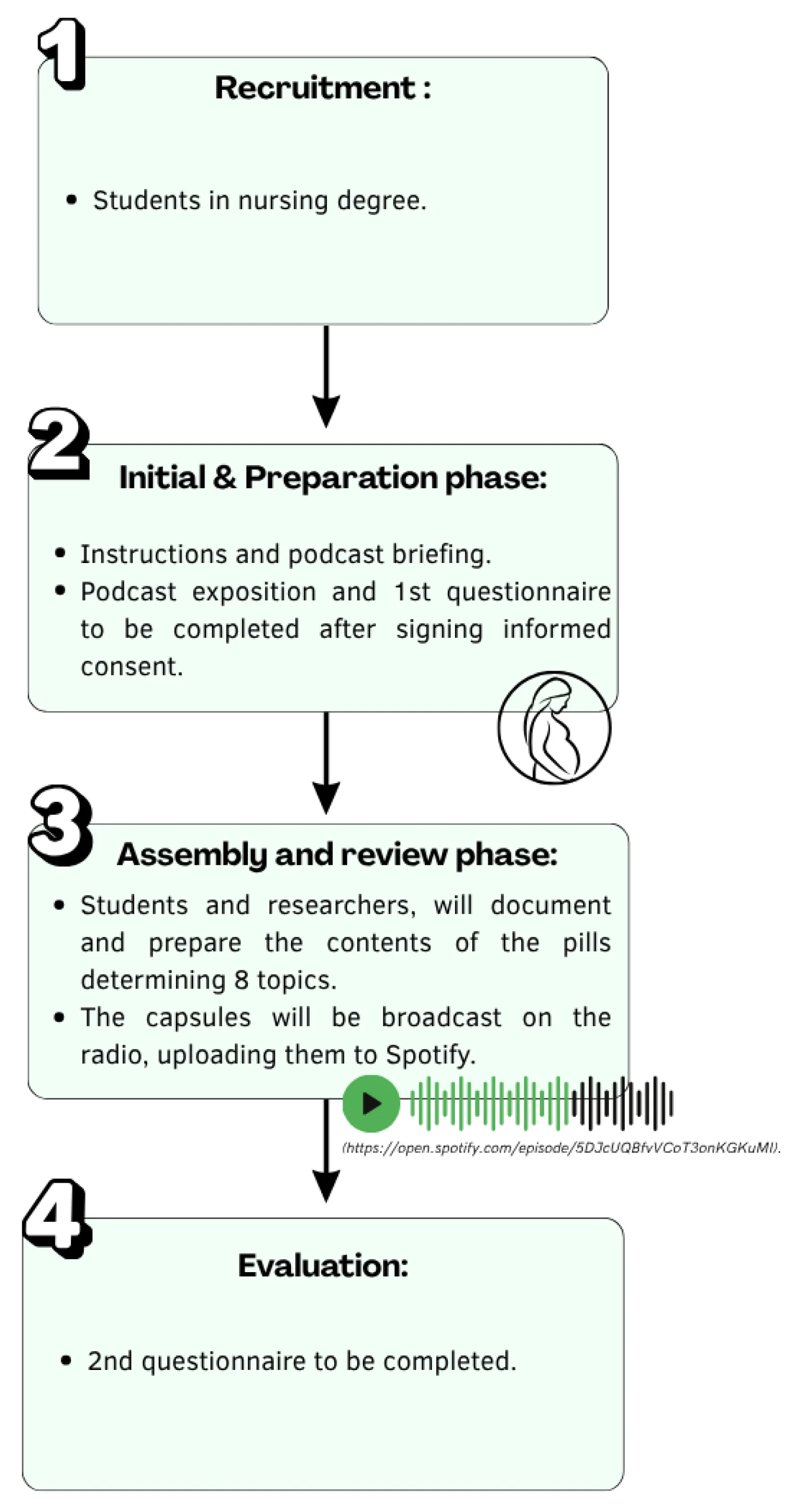
Follow-up to the educational intervention.

The number of sessions coincides with the sessions recommended by the World Health Organization and the Spanish Association of Midwives for childbirth preparation classes [6,7]. The target duration for each capsule was 5–7 min (mean realized length = 6.2 min), since various guides, protocols, and authors recommend that the duration of therapeutic advice be short [26], especially if they are podcasts and include nursing students [25]. The episodes were then reviewed and edited before being broadcast in the studio of the official radio station of the University of Jaén (UniRadio). All submitted episodes were evaluated by faculty using a rubric assessing scientific accuracy, communication clarity, narrative engagement, and technical quality. Thirty episodes achieving top rubric scores were selected to ensure that each of the eight themes was covered in at least three episodes for broadcast on the radio program Pa-ta (R) en Salud (https://open.spotify.com/episode/5DJcUQBfvVCoT3onKGKuMI) (accessed on 16 August 2026). Finally, they were asked to complete this questionnaire, created specifically for the study. The follow-up to the educational intervention using podcasts can be seen in more detail in Figure 1.

### 2.3. Information Sources

A self-administered questionnaire was created for this study, preceded by an informed consent form that all participants were required to read and accept prior to participation. The instrument was structured into three main sections. The first section collected sociodemographic data, university access pathways, academic background and motivations, home technology access, total smartphone usage, and weekly podcast consumption hours. The second section evaluated self-perceived knowledge across eight specific maternal–child health themes (pregnancy lifestyle habits, nutrition and exercise, birth plans, labor relaxation, infant feeding, postpartum sleep hygiene, support networks, and self-esteem/recreation) using a 5-point Likert scale ranging from 1 (“Very low”) to 5 (“Very high”). This section also contained a 10-item module evaluating educational satisfaction, expectation fulfilment, ease of use and production, perceived content utility/relevance for maternal health, recommendation likelihood, and future intention to produce health podcasts, rated on a 5-point Likert scale from 1 (“Completely disagree”) to 5 (“Completely agree”). Content and face validity of this ad hoc questionnaire were established by an expert panel of three university nursing professors and two clinical midwives, followed by pilot testing with a non-participating sample of 10 nursing students to ensure item clarity. The third section incorporated the Gameful Experience Scale [27], comprising 27 items across six dimensions: Enjoyment (6 items), Absorption (6 items), Creative Thinking (4 items), Activation (4 items), Absence of Negative Affect (3 items), and Mastery (4 items). Each dimension score was calculated as the mean of its constituent items, yielding a range from 1 to 5. Items 21, 22, and 23, which assess negative affect, were reverse-scored using the transformation 6—original response before calculating the Absence of Negative Affect dimension and the overall score—so that higher values consistently indicated a more favourable gameful experience. This scale was chosen because it evaluates non-traditional, intrinsically motivating psychological states (such as creative agency, cognitive immersion, and mastery) induced by active learning, moving beyond standard satisfaction metrics. Even in the absence of explicit structural gamification elements (e.g., points or badges), GAMEX effectively captures whether student-centered audio creation elicits a high-engagement, gameful mindset. The overall GAMEX score was calculated as the sum of all 27 items after reverse-scoring, with a theoretical range from 27 to 135. For all dimensions and the overall score, higher values indicate a more favourable gameful experience. The GAMEX has been validated among Spanish nursing students [28] (Márquez-Hernández et al., 2019) (demonstrating strong internal consistency with Cronbach’s alpha values ranging from 0.78 to 0.92 across dimensions) and applied in similar nursing cohorts at the University of Jaen [29] (Peinado-Molina et al., 2023). In the present sample, the overall GAMEX demonstrated high internal consistency, with Cronbach’s alpha values of 0.918 at the pre-intervention assessment and 0.945 at the post-intervention assessment.

### 2.4. Statistical Analysis

The unit of analysis was each questionnaire completed at each assessment point. Because the pre- and post-intervention questionnaires were anonymous and could not be linked at the individual level, the two assessments were analysed as independent samples.

Categorical distributions were compared using Pearson’s χ^2^ test or Fisher’s exact test when expected cell frequencies were insufficient. Ordinal self-perceived knowledge and satisfaction items, assessed using five-point Likert scales, were compared using the Mann–Whitney U test.

The six GAMEX dimension scores and the overall GAMEX score were treated as quantitative variables. Unadjusted differences between the pre- and post-intervention samples were estimated using independent-samples *t*-tests, with Welch’s correction when the assumption of equal variances was not met. Linear regression models were subsequently used to estimate adjusted mean differences, with assessment point entered as the main explanatory variable and age and sex included as covariates. Results were reported as mean differences and adjusted mean differences with their corresponding 95% confidence intervals. All statistical tests were two-sided. To control the family-wise type I error associated with multiple comparisons, the sequential Holm procedure was applied separately within three outcome families defined by the questionnaire structure: the eight self-perceived knowledge items, the ten podcast appraisal and satisfaction items, and the seven GAMEX outcomes comprising the six dimensions and the overall score. Holm adjustment was applied to the primary unadjusted between-assessment *p* values. Both unadjusted and Holm-adjusted *p* values are reported, and statistical significance was assessed using the Holm-adjusted values with a two-sided threshold of 0.05. The 95% confidence intervals are unadjusted for multiplicity and are presented as estimation measures. No missing values were present in the variables included in the reported analyses; therefore, no imputation was performed. The primary statistical analyses were performed using SPSS version 29.0.

### 2.5. Ethical Considerations

A favorable assessment was obtained from the Research Ethics Committee of the University of Jaen under reference 20240917/SEPT.PRY. All participants signed the informed consent.

## 3. Results

A total of 130 valid questionnaires were analysed at the pre-intervention assessment and 130 valid questionnaires were analysed at the post-intervention assessment. Because both assessments were anonymous and no linking code was used, it was not possible to determine how many students responded at both assessment points or to calculate individual-level attrition. Minor differences in sociodemographic characteristics between assessments reflect variations in the composition of the responding samples. The mean age in the post-intervention group was 21.47 (SD = 5.53). The majority of the students in the post-intervention group were female, with 109 (83.8%), 97 74.6% chose nursing as their first option, and 98 (75.4%) decided to enrol as a vocation. A sizable percentage, 34.6% (45), of the students spent >10 h with their smartphones. In addition, 49.2% (64) spent 1–4 h listening to podcasts online. Other sociodemographic data can be seen in Table 1.

Post-intervention respondents reported higher self-perceived knowledge across all eight maternal–child health topics. All eight comparisons remained statistically significant after Holm adjustment (all Holm-adjusted *p* < 0.001). For example, the proportion reporting high or very high self-perceived knowledge increased from 30.0% to 59.3% for pregnancy nutrition, physical activity and exercise, and from 20.7% to 53.1% for healthy and unhealthy lifestyle habits during pregnancy. The complete results are shown in Table 2.

Post-intervention respondents reported higher ratings on several podcast appraisal and satisfaction items. After Holm adjustment, general podcast satisfaction (adjusted *p* = 0.036), fulfilment of expectations (adjusted *p* = 0.036), recommendation to friends or family members (adjusted *p* < 0.001), perceived importance of podcasting for maternal health (adjusted *p* = 0.036), and perceived ease of setting up a podcast (adjusted *p* = 0.005) remained statistically significant. Recommendation to fellow nurses (adjusted *p* = 0.055) and intention to create a personal health-content podcast (adjusted *p* = 0.066) did not remain statistically significant after correction. The complete results are shown in Table 3.

Finally, the mean overall GAMEX score was 90.93 (SD = 14.50) at the pre-intervention assessment and 95.48 (SD = 18.14) at the post-intervention assessment. In the unadjusted comparisons, Creative Thinking (*p* = 0.018), Mastery (*p* = 0.047), and the overall GAMEX score (*p* = 0.026) were nominally significant. However, none remained statistically significant after Holm correction (Holm-adjusted *p* = 0.126, 0.234, and 0.158, respectively). The age- and sex-adjusted model estimated an adjusted mean difference of 4.59 points for the overall GAMEX score (95% CI 0.57 to 8.61). Because the multiplicity-adjusted inference was not statistically significant, the GAMEX findings were interpreted as exploratory. The complete results are shown in Table 4.

## 4. Discussion

The podcast-creation activity was associated with higher post-intervention self-perceived knowledge and favourable appraisal on several satisfaction items. After correction for multiple comparisons, all eight self-perceived knowledge comparisons and five of the ten podcast appraisal and satisfaction comparisons remained statistically significant. By contrast, the nominally higher Creative Thinking, Mastery, and overall GAMEX scores did not remain statistically significant after Holm adjustment. The study sample is representative of the reference population, derived from factors such as the participation of virtually all students, 92.2% of all enrolled students; the average age and the fact that the participants are predominantly female, are also similar to those of Spanish university nursing students [30,31]. Data was collected anonymously using an online questionnaire, thus allowing students to respond more honestly and avoiding possible biases from giving conditioned or socially expected answers [32,33]. On the other hand, to our knowledge, this study is the first, the first to investigate the use of podcasts for maternal and child health education, including nursing students in its design. Several limitations of this study should be considered when interpreting the findings. It is also novel and evidence remains limited. First, the single-group pre–post design lacked a control arm, which precludes making definitive causal inferences about the intervention’s efficacy, as observed changes may be influenced by confounding variables such as concurrent coursework. Second, the study was conducted at a single institution with a single cohort of first-year nursing students, limiting the generalizability of the findings to other academic levels or settings. Third, learning outcomes relied entirely on self-reported metrics rather than objective knowledge assessments (e.g., standardized examinations), introducing potential response and social desirability biases driven by voluntary survey completion. Fourth, the study lacked long-term follow-up to evaluate whether differences in self-perceived knowledge and gameful engagement are retained over time. In addition, multiple outcomes were tested; although the Holm procedure was applied within outcome families, the multiplicity-adjusted GAMEX findings were not statistically significant and should be considered exploratory. Future research should employ randomized controlled designs with multi-center cohorts, objective evaluation measures, and longitudinal assessments to validate these preliminary results.

Post-interventionrespondents reported higher levels of self-perceived knowledge than pre-intervention respondents, particularly for pregnancy nutrition and physical activity. The suggested potential utility in self-perceived knowledge skills, practice-related competencies, and confidence was also found by several authors when using podcasts as a learning method [20,21,23,24]. Furthermore, Anderson et al., [20] in their prospective study with 566 first-year nursing and midwifery students, found that designing and developing such resources can not only positively impact students’ self-perceived knowledge and attitudes, but also behavioral intentions regarding the topic treated as the podcast’s theme. Further longitudinal research is needed to determine whether these educational outcomes translate into changes in clinical practice or maternal care.

Additionally, students expressed high levels of satisfaction with podcast production. In line with our findings, Saxell et al. [34] demonstrated that positive active-learning experiences enhance core competencies, including clinical communication, problem-solving, and decision-making—skills vital for prospective healthcare professionals. While educator guidance and instructional quality significantly influence student satisfaction [35], engaging students directly as media creators provides an active, multimodal learning experience that fosters positive educational appraisals.

Furthermore, Creative Thinking, Mastery, and the composite GAMEX score were nominally higher at the post-intervention assessment, but these differences did not remain statistically significant after Holm correction. Accordingly, these GAMEX findings should be interpreted as exploratory trends rather than confirmatory evidence of enhanced gameful engagement. To our knowledge, this specific association between student-generated audio production and gameful engagement has received limited attention in the nursing education literature. In a systematic review, Tavares N. [36] observed that game-based learning strategies enhance student engagement, satisfaction, and positive learning beliefs; however, the author cautioned that time-pressured mechanics can elevate student anxiety.No statistically significant difference was found in the Absence of Negative Affect dimension between the pre- and post-intervention assessments. Our findings align with recent work by Martínez-Galiano et al. [37], who reported that applying gamified approaches to sexual and reproductive healthcare topics yielded positive effects on GAMEX dimensions among nursing undergraduates.

Finally, participants developed a stronger endorsement of digital audio media as a valuable tool for maternal health education. Previous studies evaluating podcast exposure across various nursing topics—such as mental health and biological sciences—similarly reported elevated student appreciation for audio learning [21,23,24,38]. Crucially, a distinction must be drawn between passive podcast listening and active content creation; as noted by Anderson et al. [20], the profound educational benefit stems from active involvement in scripting, producing, and broadcasting media. Students’ involvement in the design, production, and public dissemination of the podcasts may have contributed to the observed differences in self-perceived knowledge. However, differences in GAMEX scores did not remain statistically significant after Holm correction and should be considered exploratory. Podcasting is an increasingly recognized pedagogical tool in undergraduate nursing education. Building on existing literature regarding digital media, this study evaluated a theory-informed, collaboratively designed podcast-creation activity. Our findings revealed positive trends in students’ self-perceived knowledge and gameful engagement. While an uncontrolled design cannot establish causality, these results suggest that student-generated podcasting holds potential as a valuable, active-learning strategy in nursing curricula.

## 5. Conclusions

Integrating student-generated podcasting into maternal–child nursing education was associated with higher post-intervention self-perceived knowledge and favourable ratings on several satisfaction and appraisal outcomes. Higher GAMEX scores were observed in nominal analyses, but these did not remain statistically significant after Holm correction and should be interpreted as exploratory. Although an uncontrolled pre-post design precludes definitive causal claims, the activity may represent a useful active-learning approach warranting evaluation in controlled studies.

## Figures and Tables

**Table 1:** Sociodemographic characteristics of respondents at the pre- and post-intervention assessments.

Variable	Total		
	Pre-Intervention *n* (%)	Post-Intervention *n* (%)	*p* Value *
**Age** Mean (SD)	21.32 (5.51)	21.47 (5.53)	
**Sex**			0.623
Male	24 (18.5)	21 (16.2)	
Female	106 (81.5)	109 (83.8)	
**Access to Nursing Degree**			0.766
University Access	57 (43.8)	58 (44.6)	
Professional Education	66 (50.8)	63 (48.5)	
Other Degree	7 (5.4)	8 (6.2)	
Master/Postgrad	0 (0.0)	1 (0.8)	
**Nursing as 1st option**			0.887
No	34 (26.2)	33 (25.4)	
Yes	96 (73.8)	97 (74.6)	
**Other degree considered, with less clinical presence**			0.602
No	109 (83.8)	112 (86.2)	
Yes	21 (16.2)	18 (13.8)	
Master’s Degree	-	-	
**Motivations to study Nursing**			0.817
Professional opportunities	16 (12.3)	18 (13.8)	
Vocational	95 (73.1)	98 (75.4)	
International opportunities	3 (2.3)	1 (0.8)	
1st option not achievable	9 (6.9)	8 (6.2)	
Other	7 (5.4)	5 (3.8)	
**All subjects passed**			0.690
No	40 (30.8)	43 (33.1)	
Yes	90 (69.2)	87 (66.9)	
**Computer at home**			1.000
No	1 (0.8)	1 (0.8)	
Yes	129 (99.2)	129 (99.2)	
**Internet Access at home**			0.316
No	1 (0.8)	0 (0.0)	
Yes	129 (99.2)	130 (100.0)	
**Weekly hours on smartphone (using internet, among others)**			0.650
0 h	0 (0.0)	0 (0.0)	
1–4 h	34 (26.2)	40 (30.8)	
5–10 h	45 (34.6)	45 (34.6)	
>10 h	51 (39.2)	45 (34.6)	
**From these hours on the internet, how much time is spent listening to podcasts?**			0.081
0 h	76 (58.5)	59 (45.4)	
1–4 h	48 (36.9)	64 (49.2)	
5–10 h	5 (3.8)	3 (2.3)	
>10 h	1 (0.8)	4 (3.1)	

* Pearson’s chi-squared (χ^2^) test.

**Table 2:** Knowledge of the topic and self-assessment, preparing the podcast before and after the intervention.

Topic and Self-Perceived Knowledge Evaluation	Pre-Intervention *n* (%)	Post-Intervention *n* (%)	Pre, Mean (DE)	Post, Mean (DE)	Unadjusted *p* Value *	Holm-Adjusted *p* Value
**Topic 1. Pregnancy: Healthy and Unhealthy Lifestyle Habits**			2.94 (0.82)	3.61 (0.78)	<0.001	<0.001
Very low	9 (6.9)	0 (0.0)				
Low	19 (14.6)	7 (5.4)				
Average	75 (57.7)	54 (41.5)				
High	25 (19.2)	52 (40.0)				
Very high	2 (1.5)	17 (13.1)				
**Topic 2. Pregnancy: Proper and Balanced Nutrition. Physical Activity and Exercise**			3.03 (0.94)	3.72 (0.78)	<0.001	<0.001
Very low	10 (7.7)	0 (0.0)				
Low	20 (15.4)	5 (3.8)				
Average	61 (46.9)	48 (36.9)				
High	34 (26.2)	56 (43.1)				
Very high	5 (3.8)	21 (16.2)				
**Topic 3. Childbirth: Expectations and Birth Plan**			2.20 (0.80)	3.26 (0.97)	<0.001	<0.001
Very low	23 (17.7)	1 (0.8)				
Low	65 (50.0)	28 (21.5)				
Average	36 (27.7)	54 (41.5)				
High	5 (3.8)	30 (23.1)				
Very high	1 (0.8)	17 (13.1)				
**Topic 4. Childbirth: Relaxation**			2.23 (0.97)	3.30 (0.95)	<0.001	<0.001
Very low	33 (25.4)	2 (1.5)				
Low	49 (37.7)	23 (17.7)				
Average	34 (26.2)	54 (41.5)				
High	13 (10.0)	36 (27.7)				
Very high	1 (0.8)	15 (11.5)				
**Topic 5. Postpartum: Expectations and Baby Feeding**			2.20 (0.87)	3.27 (0.93)	<0.001	<0.001
Very low	25 (19.2)	2 (1.5)				
Low	65 (50.0)	25 (19.2)				
Average	31 (23.8)	51 (39.2)				
High	7 (5.4)	40 (30.8)				
Very high	2 (1.5)	12 (9.2)				
**Topic 6. Postpartum: Sleep**			2.21 (0.90)	3.13 (0.94)	<0.001	<0.001
Very low	28 (21.5)	3 (2.3)				
Low	60 (46.2)	30 (23.1)				
Average	30 (23.1)	55 (42.3)				
High	11 (8.5)	31 (23.8)				
Very high	1 (0.8)	11 (8.5)				
**Topic 7. Parenting: Support Network: Family and Partner**			2.82 (0.93)	3.41 (0.94)	<0.001	<0.001
Very low	12 (9.2)	2 (1.5)				
Low	31 (23.8)	19 (14.6)				
Average	59 (45.4)	49 (37.7)				
High	25 (19.2)	44 (33.8)				
Very high	3 (2.3)	16 (12.3)				
**Topic 8. Parenting: Self-Esteem and Personal Recreation**			2.68 (1.04)	3.24 (0.92)	<0.001	<0.001
Very low	20 (15.4)	3 (2.3)				
Low	35 (26.9)	24 (18.5)				
Average	45 (34.6)	52 (40.0)				
High	27 (20.8)	41 (31.5)				
Very high	3 (2.3)	10 (7.7)				

* Unadjusted *p* values were obtained using the Mann–Whitney U test for independent samples. Holm-adjusted *p* values were calculated using the sequential Holm procedure within the corresponding outcome family. Means and standard deviations are presented as descriptive statistics.

**Table 3:** Podcast Evaluation before and after the intervention.

Podcast Self-Assessment and Satisfaction	Pre-Intervention *n* (%)	Post-Intervention *n* (%)	Pre, Mean (DE)	Post, Mean (DE)	Unadjusted *p* Value *	Holm-Adjusted *p* Value
**Podcast satisfaction**			4.20 (0.68)	4.41 (0.70)	0.006	0.036
Completely disagree	0 (0.0)	1 (0.8)				
Disagree	3 (2.3)	0 (0.0)				
Neither agree nor disagree	10 (7.7)	10 (7.7)				
Agree	75 (57.7)	53 (40.8)				
Completely agree	42 (32.3)	66 (50.8)				
**The podcast met expectations**			4.17 (0.65)	4.38 (0.70)	0.005	0.036
Completely disagree	0 (0.0)	1 (0.8)				
Disagree	3 (2.3)	0 (0.0)				
Neither agree nor disagree	9 (6.9)	10 (7.7)				
Agree	81 (62.3)	57 (43.8)				
Completely agree	37 (28.5)	62 (47.7)				
**I would recommend a friend or family member to participate**			3.93 (0.86)	4.34 (0.80)	<0.001	<0.001
Completely disagree	1 (0.8)	2 (1.5)				
Disagree	1 (0.8)	1 (0.8)				
Neither agree nor disagree	43 (33.1)	12 (9.2)				
Agree	46 (35.4)	51 (39.2)				
Completely agree	39 (30.0)	64 (49.2)				
**I would recommend participation to a fellow nurse**			4.32 (0.76)	4.52 (0.77)	0.011	0.055
Completely disagree	1 (0.8)	2 (1.5)				
Disagree	0 (0.0)	1 (0.8)				
Neither agree nor disagree	17 (13.1)	7 (5.4)				
Agree	50 (38.5)	38 (29.2)				
Completely agree	62 (47.7)	82 (63.1)				
**Using the podcast and its mobile app is easy**			4.55 (0.76)	4.51 (0.70)	0.315	0.315
Completely disagree	2 (1.5)	1 (0.8)				
Disagree	1 (0.8)	1 (0.8)				
Neither agree nor disagree	6 (4.6)	6 (4.6)				
Agree	35 (26.9)	45 (34.6)				
Completely agree	86 (66.2)	77 (59.2)				
**The content answers important questions**			4.44 (0.65)	4.55 (0.65)	0.098	0.256
Completely disagree	0 (0.0)	1 (0.8)				
Disagree	1 (0.8)	1 (0.8)				
Neither agree nor disagree	8 (6.2)	2 (1.5)				
Agree	54 (41.5)	47 (36.2)				
Completely agree	67 (51.5)	79 (60.8)				
**The content is relevant**			4.49 (0.65)	4.62 (0.60)	0.085	0.256
Completely disagree	0 (0.0)	1 (0.8)				
Disagree	1 (0.8)	0 (0.0)				
Neither agree nor disagree	8 (6.2)	2 (1.5)				
Agree	47 (36.2)	41 (31.5)				
Completely agree	74 (56.9)	86 (66.2)				
**The podcast is essential for maternal health**			3.87 (0.85)	4.16 (0.81)	0.004	0.036
Completely disagree	0 (0.0)	1 (0.8)				
Disagree	7 (5.4)	1 (0.8)				
Neither agree nor disagree	35 (26.9)	25 (19.2)				
Agree	56 (43.1)	52 (40.0)				
Completely agree	32 (24.6)	51 (39.2)				
**Setting up a podcast is very easy**			2.75 (1.20)	3.27 (1.22)	0.001	0.005
Completely disagree	20 (15.4)	10 (7.7)				
Disagree	40 (30.8)	28 (21.5)				
Neither agree nor disagree	36 (27.7)	34 (26.2)				
Agree	21 (16.2)	33 (25.4)				
Completely agree	13 (10.0)	25 (19.2)				
**I’m considering creating my own health content podcast**			2.37 (0.98)	2.75 (1.21)	0.017	0.066
Completely disagree	28 (21.5)	21 (16.2)				
Disagree	42 (32.3)	37 (28.5)				
Neither agree nor disagree	47 (36.2)	40 (30.8)				
Agree	10 (7.7)	17 (13.1)				
Completely agree	3 (2.3)	15 (11.5)				

* Unadjusted *p* values were obtained using the Mann–Whitney U test for independent samples. Holm-adjusted *p* values were calculated using the sequential Holm procedure within the corresponding outcome family. Means and standard deviations are presented as descriptive statistics.

**Table 4:** Evolution of the GAMEX scores and their pre-post dimensions.

GAMEX Score	Total		Bivariate Analysis/Regression			Multivariate Analysis *	Holm-Adjusted *p*
	Pre-Intervention Mean (SD)	Post-Intervention Mean (SD)	Unadjusted *p*	MD	(95% CI)	aMD (95% CI)	
**Enjoyment**	3.73 (0.66)	3.91 (0.84)	0.069	0.18	(−0.01, 0.35)	0.17 (−0.01, 0.36)	0.257
**Absorption**	3.00 (0.86)	3.19 (1.07)	0.106	0.19	(−0.04, 0.43)	0.19 (−0.05, 0.43)	0.257
**Creative thinking**	3.47 (0.87)	3.73 (0.88)	0.018	0.25	(0.05, 0.47)	0.26 (0.05, 0.48)	0.126
**Activation**	3.05 (0.75)	3.24 (0.88)	0.064	0.19	(−0.01, 0.39)	0.19 (−0.01, 0.39)	0.257
**Absence of** **Negative Affect**	4.06 (0.82)	3.99 (1.07)	0.530	−0.07	(−0.30, 0.15)	−0.07 (−0.31, 0.16)	0.530
**Mastery**	3.05 (0.78)	3.24 (0.78)	0.047	0.19	(0.01, 0.39)	0.20 (0.10, 0.39)	0.234
**GAMEX total**	90.93 (14.50)	95.48 (18.14)	0.026	4.54	(0.54, 8.56)	4.59 (0.57, 8.61)	0.158

MD, mean difference; aMD, adjusted mean difference; CI, confidence interval. Unadjusted comparisons were performed using independent-samples *t*-tests, with Welch’s correction when appropriate. Holm-adjusted *p* values were calculated across the six GAMEX dimensions and the overall GAMEX score. * Adjusted mean differences were estimated using linear regression models adjusted for age and sex. Confidence intervals are unadjusted for multiplicity and are presented for estimation; statistical significance was assessed using Holm-adjusted *p* values.

## Data Availability

The data presented in this study are available on request from the corresponding author.

## Public Involvement Statement

No public involvement in any aspect of this research.

## Guidelines and Standards Statement

This manuscript was drafted in accordance with the Transparent Reporting of Evaluations with Nonrandomized Designs (TREND) statement for the reporting of a nonrandomized educational intervention study.
